# Association of Leisure-Time Physical Activity and Mortality Risk in High Cardiovascular Risk Population with and without Left Ventricular Hypertrophy 

**DOI:** 10.31083/j.rcm2410285

**Published:** 2023-10-08

**Authors:** Huijun Jin, Xiulin Wang, Hao Dai, Aoxi Tian, Bowang Chen, Chaoqun Wu, Xiaoyan Zhang, Jianlan Cui, Yi Wu, Xi Li, Xin Zheng

**Affiliations:** ^1^National Clinical Research Center for Cardiovascular Diseases, State Key Laboratory of Cardiovascular Disease, Fuwai Hospital, National Center for Cardiovascular Diseases, Chinese Academy of Medical Sciences and Peking Union Medical College, 100037 Beijing, China; ^2^Department of Cardiology, Xuanwu Hospital, Capital Medical University, 100053 Beijing, China; ^3^Central China Sub-Center of the National Center for Cardiovascular Diseases, 450000 Zhengzhou, Henan, China; ^4^National Clinical Research Center for Cardiovascular Diseases, Shenzhen, Coronary Artery Disease Center, Fuwai Hospital Chinese Academy of Medical Sciences Shenzhen, 518057 Shenzhen, Guangdong, China

**Keywords:** leisure-time physical activity, left ventricular hypertrophy, cardiac remodeling, cardiovascular risks factors, metabolic equivalent

## Abstract

**Background:**

Increased leisure-time physical activity (LTPA) is linked with decreased mortality risk, while also with increased left ventricular mass, which may induce left ventricular hypertrophy (LVH). We investigated whether LVH modifies the association between higher LTPA and lower mortality risk in population at high cardiovascular risk.

**Methods:**

In a prospective national cohort, we used the left ventricular mass/body surface area (LVM/BSA) method to define LVH. Baseline LTPA was self-reported and divided into: low (<500 metabolic equivalent of task [MET]) min/week), moderate (500–1999 MET min/week) and high (≥2000 MET-min/week). Analyses of the dose-response relationship between LTPA and left ventricular mass were performed using restricted cubic spline regression. A multivariate adjusted Cox proportional hazards regression analysis was used to estimate hazard ratios (HRs).

**Results:**

A total of 163,006 participants (55.3% females, mean [standard deviation] age, 62.4 [7.4] years) were included. During a median of 4.8 years of follow-up, 6586 (4.0%) died from all causes and 3024 (1.9%) from cardiovascular causes. Multivariate adjusted Cox proportional hazards regression analyses revealed that moderate and high LTPA were linked with less cardiovascular and all-cause mortality risk than low LTPA in the absence of LVH. In those with LVH, the association of high (0.83, 0.69–0.99) or moderate (0.72, 0.56–0.91) LTPA with cardiovascular mortality risk persisted. For all-cause mortality risk, this association was only significant in high LTPA (0.73, 0.61–0.86), while marginal in moderate LTPA (0.96, 0.84 to 1.08). Overall, the correlation patterns between LTPA and mortality risk appears distinct between those with LVH and those without LVH; the modification of LVH was not significant regarding mortality risk among the high cardiovascular risk population (all-cause: *p*-value for interaction = 0.074; cardiovascular cause: *p*-value for interaction = 0.581), except in females regarding all-cause mortality risk (*p*-value for interaction = 0.006).

**Conclusions:**

The association between higher LTPA and lower mortality risk was not modified by LVH in high cardiovascular risk population. However, the presence of LVH altered this association in females regarding the all-cause mortality risk.

## 1. Introduction

Regular leisure-time physical activity (LTPA) of at least 500 to 1000 metabolic equivalent of task (MET)-minutes per week without upper limits is consistently advised in patients at high cardiovascular risk [1], based on the evidence of its advantages in lowering the incidence and mortality of cardiovascular disease (CVD) [2]. Nevertheless, in this population, left ventricular hypertrophy (LVH) is a prevalent condition related to poor prognosis, and has a complicated relationship with LTPA at different levels [3, 4, 5]. Several studies have shown that moderate LTPA intensity and duration was related to a reduced risk of LVH and might produce regression of LVH among high-cardiovascular risk patients [6, 7, 8]. In contrast, some studies found that strenuous physical activity was linked with increased left ventricular mass, which may induce hypertrophy, moreover increase the risk of death [9, 10, 11, 12]. In healthy individuals, exercise is commonly linked with benign and reversible cardiac remodeling [9]. Nonetheless, in individuals with certain predisposing risk factors such as electrical cardiac abnormalities and pathological hypertrophy, etc., high-intensity exercise might be linked with an unfavorable prognosis and increased risk of mortality [13]. High-risk cardiovascular populations exhibited multiple risk factors, including hypertension, diabetes, etc., which might contribute to electrical cardiac remodeling, myocardial fibrosis, and pathological hypertrophy [14].

In this case, the influence of LVH on the dose-response association between LTPA and mortality risk in high-risk cardiovascular populations remains unknown. In other words, whether the benefits of moderate to high volumes of LTPA regarding mortality risk could be modified by the presence of LVH has not been extensively studied. This is critical for providing tailored prevention measures, particularly in high cardiovascular risk population.

Therefore, we analyzed the data from a national population-based cohort, with standardized echocardiography conducted in individuals with high cardiovascular risk at baseline. We aimed to evaluate whether the existence of LVH alters the associations between LTPA levels and all-cause and cardiovascular mortality risks.

## 2. Materials and Methods

### 2.1 Study Design and Participants

The China PEACE (Patient-centered Evaluative Assessment of Cardiac Events) Million People Project, a government-funded public health initiative, was launched in 2014. The project design has been previously explained in full [15]. Research sites were chosen from each of 31 provinces in China to ensure a diversified geographical distribution, population structure, exposure to risk variables, and disease trends (**Supplementary Methods**).

In this project, community residents aged 35 to 75 years old with a residency history of at least six out of the preceding twelve months were recruited. Questionnaire interviews, physical measurements, and laboratory testing were conducted to obtain information on socioeconomic characteristics, health status, risk factors, and drug use. In individuals with high CVD risk defined based on their medical history and risk factor profile, echocardiography and carotid ultrasound were also performed.

In the present study, we included individuals with an assessed 10-year CVD risk of greater than 10% (according to factors including age, gender, blood pressure, diabetes, total cholesterol, body mass index (BMI), and smoking status), based on the 2019 CVD risk charts of the World Health Organization [16] (**Supplementary Methods**), and those who underwent routine echocardiography. The individuals with previous episodes of CVD or stroke were excluded.

The research was approved by China’s National Center for Cardiovascular Diseases Ethics Council, which is located in Beijing. All participants provided their written informed consent.

### 2.2 LTPA

LTPA was evaluated with a verified questionnaire [17], which included questions to collect information about the type, frequency, and duration of activity performed during the past 12 months. Tai Chi/qigong/leisure walking, swimming, jogging/aerobic exercise, brisk walking/gymnastics/folk dance, ball games, mountain hiking, home exercise, and rope jumping were presented examples of various activities. Based on the 2011 Compendium of Physical Activity, each LTPA was allocated a unique metabolic equivalent of task (MET) [18]. Frequency, intensity, and duration were added together to get the energy expenditure (MET-min/week) associated with LTPA. Low (<500 MET-min/week), moderate (500–1999 MET-min/week), and high (≥2000 MET-min/week) amounts of LTPA were identified [19].

### 2.3 Echocardiogram

All echocardiography ultrasound examinations were performed according to a standardized protocol by certificated ultrasound physicians. Before the implementation of this project, a quality control team comprised of senior ultrasound physicians from the National Center for Cardiovascular Diseases had monitored compliance and quality of ultrasound examinations at each study site. During the implementation, echocardiograms ultrasound images were transmitted to the National Center for Cardiovascular Diseases in DICOM format (JPG and AVI from rural sites where DICOM files were not available) for central adjudication.

End-diastolic ultrasound measurements included the thickness of the posterior wall, end-diastolic diameter, and septal wall thickness of the left ventricle. Using the Devereux formula, the left ventricular mass index (LVMI) was determined by dividing the anatomic mass by the body surface [20]. According to American Society of Echocardiography (ASE)/European Association for Cardiovascular Imaging (EACI) guidelines, LVH was defined as LVMI of 115 ${\mathrm{g/m}}^{2}$ or higher in males and 95 ${\mathrm{g/m}}^{2}$ or higher in females [21].

### 2.4 Ascertainment of Outcomes

All analyses utilized data accessible through December 31, 2021. Using the National Mortality Surveillance System and Vital Registration of China’s Center for Disease Prevention and Control, we determined each participant’s vital status and cause of death. We obtained confirmation of death from local residential, medical, and health insurance records. The 10th Edition of the International Classification of Diseases (ICD) was used to categorize the underlying causes of death. All-cause and cardiovascular mortality were the primary health outcomes (**Supplementary Methods**).

### 2.5 Statistical Analysis

Continuous variables were reported as mean (standard deviation, SD) or medians (inter quartile range, IQR), and categorical variables as number (percent). One-way ANOVA was used to evaluate continuous variables. Categorical variables were compared using the χ^2^ test. The connection between LTPA and LVMI was analyzed using generalized linear regression models. We introduced the median value of each LTPA group as a continuous variable in the models and tested for linear trend [22]. Cox proportional hazard models were utilized to construct adjusted hazard ratios (HRs) and 95% confidence intervals (CIs), with study sites serving as a random effect to account for the clustering of people for all-cause mortality risk. For cardiovascular mortality risk, a Fine and Grey sub-distribution hazards model was executed, incorporating death from other causes as a competing risk. Age, gender, BMI, hypertension, diabetes, dyslipidemia, smoking, alcohol use, education levels, income levels, residential region (rural or urban), and the use of anti-diabetic, anti-hypertensive, and statin drugs were considered as confounding variables. The interaction between LTPA and LVH was considered in these models. To address the issue of reverse causality, sensitivity analyses excluding death in the first six months after the baseline period were conducted.

The analyses were performed by SAS 9.4 (SAS Institute Inc., Cary, NC, USA) and statistical significance was defined as a two-sided *p*-value < 0.05.

## 3. Results

### 3.1 Baseline Characteristics

The data of 195,567 participants with a high cardiovascular risk and available echocardiograms were analysed. After excluding those with missing information on LTPA (3584, 1.8%), and those with a history of CVDs (28,977, 15.1%), 163,006 were included (**Supplementary Fig. 1**). At baseline, the mean (SD) age was 62.4 (7.4) years, and 90078 (55.3%) were females (Table 1). In total, 89.4% had a history of hypertension, 35.0% diabetes, 55.9% dyslipidemia, and 41.3% obesity; while 21.5% and 20.4% were current tobacco smokers and alcohol drinkers, respectively. In addition, 65.2% of the population resided in rural areas, 14.1% had a family income of above 7000 USD per year, and 17.2% had at least a high school diploma.

**Table 1.** **Baseline characteristics**.

Variables		Overall	Non-LVH	LVH	*p*-value*
		(n = 163,006)	(n = 120,234)	(n = 42,772)	
Age (years), mean (SD)		62.4 (7.4)	58.6 (8.8)	63.8 (6.6)	<0.001
Age (years), *n* (%)					
	35–44	3719 (2.3)	3370 (2.8)	349 (0.8)	
	45–54	20,815 (12.8)	17,238 (14.3)	3577 (8.4)	
	55–64	69,015 (42.4)	51,357 (42.8)	17,658 (41.2)	
	65–75	69,457 (42.6)	48,269 (40.1)	21,188 (49.5)	
Female sex, *n* (%)		90,078 (55.3)	56,655 (47.1)	33,423 (78.1)	<0.001
Education, *n* (%)					
	Primary school or lower	88,157 (54.1)	60,028 (49.9)	28,129 (65.8)	<0.001
	Middle school	46,803 (28.7)	37,012 (30.8)	9791 (22.9)	<0.001
	High school	20,609 (12.6)	16,807 (14.0)	3802 (8.9)	<0.001
	College or above	7437 (4.6)	6387 (5.3)	1050 (2.5)	<0.001
Household income (USD/year), *n* (%)					
	<1400	41,593 (25.5)	28,350 (23.6)	13,243 (31.0)	<0.001
	1400–7000	98,441 (60.4)	74,042 (61.6)	24,399 (57.0)	<0.001
	≥7000	22,972 (14.1)	17,842 (14.8)	5130 (12.0)	<0.001
Urbanity, *n* (%)					
	Rural	108,656 (65.2)	79,303 (64.4)	29,353 (67.5)	<0.001
	Urban	54,349 (34.8)	40,932 (35.6)	13,419 (32.5)	<0.001
Metabolic factors, *n* (%)					
	Hypertension ^a^	145,665 (89.4)	105,750 (88.0)	39,975 (93.3)	<0.001
	Diabetes ^b^	57,111 (35.0)	42,516 (35.4)	14,595 (34.1)	<0.001
	Dyslipidemia ^c^	91,122 (55.9)	67,758 (56.4)	23,364 (54.6)	<0.001
	Obesity ^d^	67,283 (41.3)	54,475 (45.3)	12,808 (29.9)	<0.001
Lifestyle, *n* (%)					
	Tobacco smoker	35,091 (21.5)	30,552 (25.4)	4539 (10.6)	<0.001
	Alcohol drinker	33,189 (20.4)	28,503 (23.7)	4686 (11.0)	<0.001
LTPA, *n* (%)					
	Low LTPA	107,407 (65.9)	77,663 (64.6)	29,744 (69.5)	<0.001
	Moderate LTPA	36,047 (22.1)	27,763 (23.1)	8284 (19.4)	<0.001
	High LTPA	19,552 (12.0)	14,808 (12.3)	4744 (11.1)	<0.001
LVMI, ${\mathrm{g/m}}^{2}$		88.3 (75.1, 103.3)	81.5 (71.1, 91.2)	113.8 (102.7, 124.9)	<0.001

Data presented as number n proportion (%) or mean (SD). Abbreviations: LVH, left ventricular hypertrophy; LVMI, left ventricular mass index; LTPA, leisure time physical activity; MET, metabolic equivalent of task; Low LTPA, <500 MET-min/week; Moderate LTPA, 500–1999 MET-min/week; High LTPA, ≥2000 MET-min/week; FBG, fasting blood glucose; SBP, systolic blood pressure; DBP, diastolic blood pressure; BMI, body mass index; TG, triglyceride; TC, total cholesterol; LDL-C, low density lipoprotein cholesterol; HDL-C, high density lipoprotein cholesterol. * *p*-value indicate the comparison between Non-LVH and LVH groups. ^a^ Self-reported use of antihypertensive medications, SBP ≥140 mmHg, and/or DBP ≥90 mmHg were used to determine hypertension. ^b^ Self-reported use of antidiabetic medications or FBG ≥7.0 mmol/L were used to define diabetes. ^c^ Self-reported use of lipid-lowering drugs or TC ≥5.2 mmol/L, LDL-C ≥3.4 mmol/L, HDL-C ≤1.0 mmol/L and/or TG ≥1.7 mmol/L were used to indicate dyslipidemia. ^d^ Obesity was determined based on BMI ≥28 ${\mathrm{kg/m}}^{2}$.

Overall, LVH was present in 26.2% of the study population. Compared to those without LVH, individuals with LVH were more prone to be female, older, and rural residents, and had less advanced education and income. In addition, the LVH group has higher frequency of hypertension (93.3% versus 88.0%, *p*-value < 0.001). Low LTPA was more prevalent among those with LVH compared to the population without LVH (69.5% versus 64.6%, *p*-value < 0.001).

### 3.2 LTPA and LVMI 

**Fig. 1.**
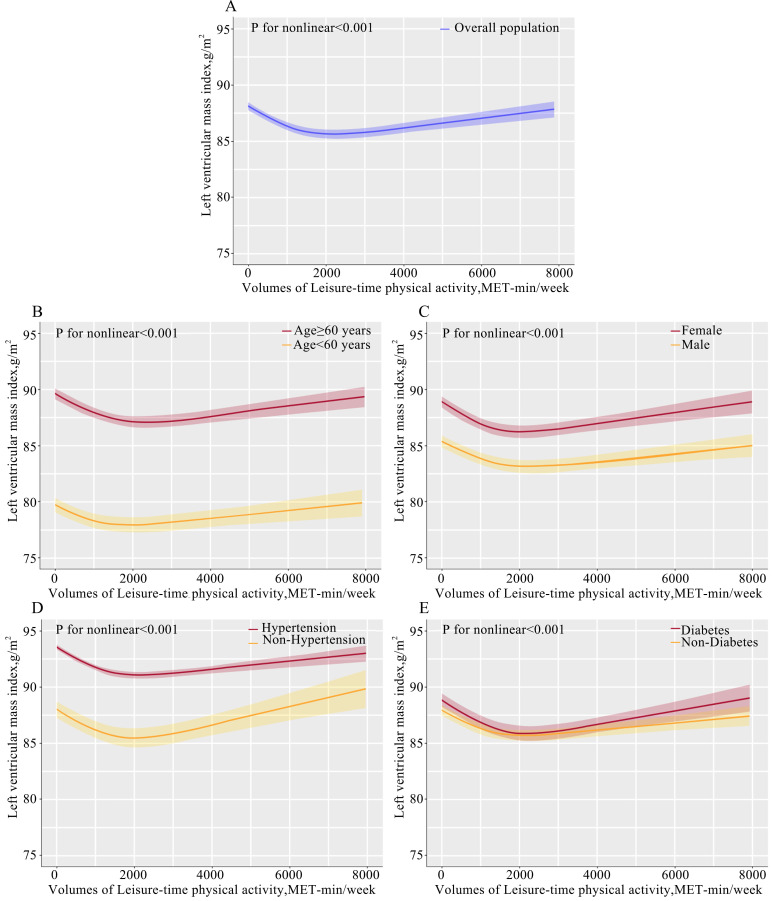
**Dose-response associations between leisure-time physical activity and LVMI.** The dose-association between LVMI and leisure-time physical activity was performed in whole population (A) and in the subgroup stratified by of age (B), sex (C), hypertension (D), and diabetes (E). Abbreviations: LVMI, left ventricular mass index; MET, metabolic equivalent of task; BMI, body mass index. *p*-value for nonlinear indicates that the dose-relationship between LVMI and leisure-time physical activity using restricted cubic spline curves adjusted for confounders of age, sex, BMI, hypertension, dyslipidemia, diabetes, smoking status, drinking, education levels, income levels, and medication use.

Using a multivariable restricted cubic spline regression, the curvilinear pattern was observed in the association of volumes of LTPA and LVMI after adjusting for age, sex, BMI, hypertension, dyslipidemia, diabetes, smoking, drinking, education, income, and medication use (Fig. 1). In comparison with low LTPA, there was an incrementally lower LVMI until leveling occurred at approximately 2000 in the whole population, and LVMI showed a slow increase after a threshold of approximately 2000, which indicated that the high LTPA group (≥2000 MET-min/week) was linked to increased LVMI. Furthermore, we also examined this dose-relationship in the subgroups stratified by age, sex, hypertension, and diabetes, and had similar observations in the whole population (Fig. 1).

### 3.3 LTPA and Mortality Risk

During a median of 4.8 years of follow-up, 6586 (4.0%) all-cause deaths and 3024 (1.9%) cardiovascular deaths occurred in the study population. Specifically, 4773 (4.44%) and 2270 (2.11%) in the low LTPA group, 1173 (3.25%) and 480 (1.33%) in the moderate LTPA group, and 640 (3.27%) and 274 (1.40%) in the high LTPA group, respectively (Table 2, **Supplementary Fig. 2**).

**Table 2.** **LTPA and all-cause and cardiovascular mortality risks stratified by the presence of LVH**.

Volumes of LTPA (MET-min/week)		All-cause mortality			Cardiovascular mortality		
		Rates, % (N)	Unadjusted	Adjusted	Rates, % (N)	Unadjusted	Adjusted
			HR (95% CI)	HR (95% CI) *		HR (95% CI)	HR (95% CI) *
Non-LVH		3.83% (4601)			1.67% (2011)		
	Low (<500)	4.23% (3286)	Reference	Reference	1.91% (1485)	Reference	Reference
	Moderate (500–1999)	3.01% (837)	0.77 (0.71–0.83)	0.80 (0.73–0.86)	1.19% (331)	0.66 (0.59–0.74)	0.71 (0.63–0.81)
	High (≥2000)	3.23% (478)	0.76 (0.69–0.84)	0.77 (0.70–0.85)	1.32% (195)	0.69 (0.60–0.81)	0.73 (0.62–0.85)
LVH		4.64% (1985)			2.37% (1013)		
	Low (<500)	5.00% (1487)	Reference	Reference	2.64% (785)	Reference	Reference
	Moderate (500–1999)	4.06% (336)	0.88 (0.78–0.99)	0.96 (0.84–1.08)	1.80% (149)	0.72 (0.60–0.85)	0.83 (0.69–0.99)
	High (≥2000)	3.41% (162)	0.66 (0.56–0.78)	0.73 (0.61–0.86)	1.67% (79)	0.62 (0.49–0.78)	0.72 (0.56–0.91)

Abbreviations: LVH, left ventricular hypertrophy; LTPA, leisure-time physical activity; MET, metabolic equivalent of task; HR, hazard ratio; CI, confidence interval; BMI, body mass index. * Multivariable-adjusted model was adjusted for age, sex, BMI, hypertension, dyslipidemia, diabetes, smoking, drinking, education levels, income levels, sites, and medication use. The interaction effect of LTPA and LVH was *p* for interaction = 0.074 for all-cause mortality; *p* for interaction = 0.581 for cardiovascular mortality.

**Fig. 2.**
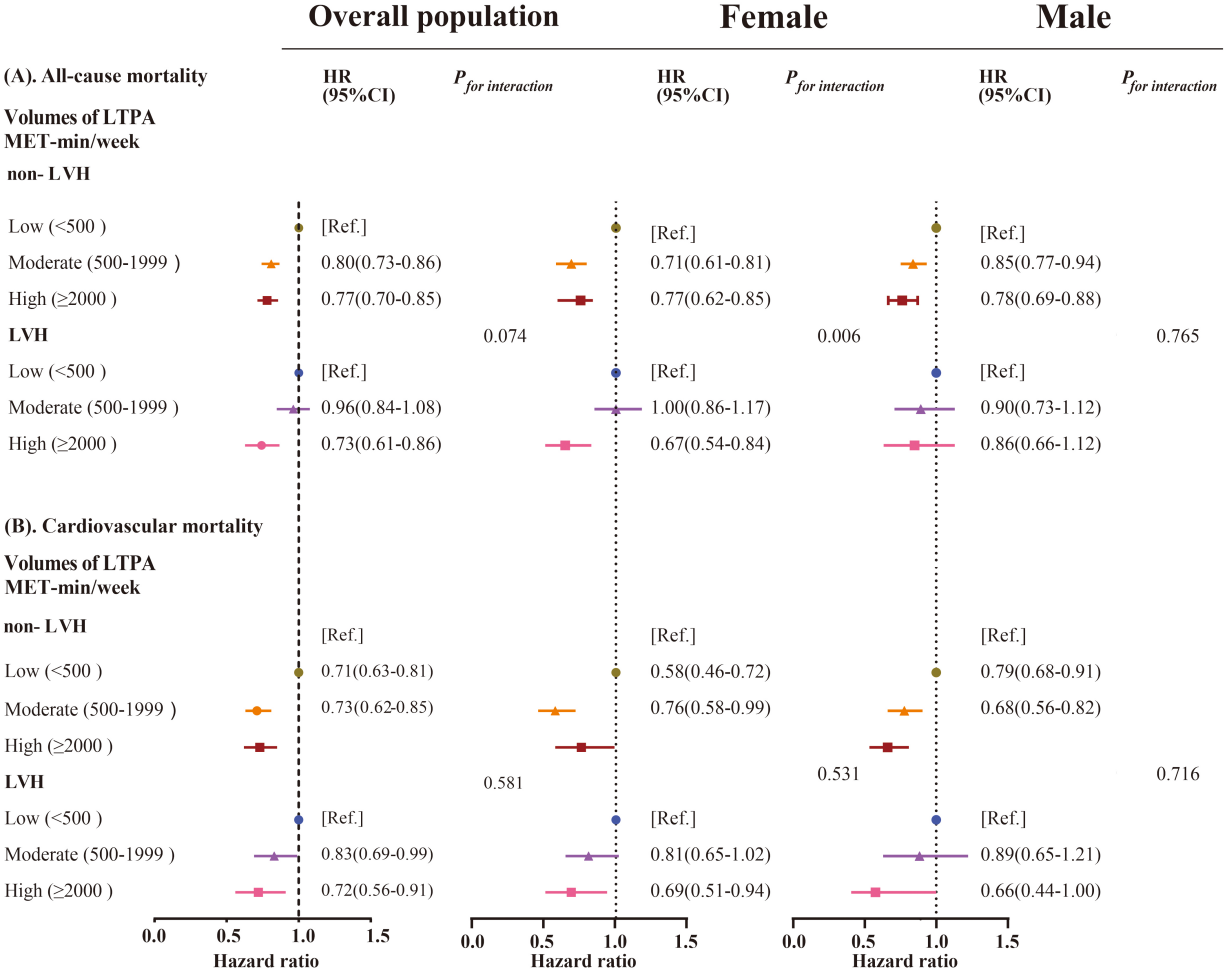
**LTPA and the risk of mortality stratified by LVH in males and females.** (A) LTPA and the risk of all-cause mortality in overall population and stratified by males and females. (B) LTPA and the risk of cardiovascular mortality in overall population and stratified by males and females. Abbreviations: LVH, left ventricular hypertrophy; LTPA, leisure-time physical activity; HR, hazard ratio (95% CI); CI, confidence interval; MET, metabolic equivalent of task; BMI, body mass index. Multivariable adjusted model was adjusted for age, sex, BMI, hypertension, dyslipidemia, diabetes, smoking, drinking, education levels, income levels, sites, and medication use. The interaction effect of LTPA and LVH on all-cause mortality: *p*-value for interaction = 0.074 in whole population; *p*-value for interaction = 0.765 in males; *p*-value for interaction = 0.006 in females; cardiovascular mortality: *p*-value for interaction = 0.581 in whole population; *p*-value for interaction = 0.716 in males; *p*-value for interaction = 0.531 in females.

Among the participants without LVH, those with a high LTPA had lower all-cause mortality [adjusted HR = 0.77 (0.70–0.85)] and cardiovascular mortality [adjusted HR = 0.73 (0.62–0.85)] compared to those with a low LTPA; while for individuals with moderate LTPA, the adjusted HRs were 0.80 (0.73–0.86) and 0.71 (0.63–0.81) for all-cause mortality and cardiovascular mortality, respectively (Table 2, Fig. 2).

In participants with LVH, high LTPA was independently linked with lower mortality, with adjusted HR of 0.73 (0.61–0.86) for all-cause mortality and 0.72 (0.56–0.91) for cardiovascular mortality. However, moderate LTPA showed no significant effect on reducing all-cause mortality (0.96 [0.84–1.08]), but the benefits were observed in cardiovascular mortality (0.83 [0.69–0.99]) (Table 2, Fig. 2). The association was not modified by LVH for all-cause deaths (*p*-value for interaction = 0.074) or cardiovascular deaths (*p*-value for interaction = 0.581).

**Fig. 3.**
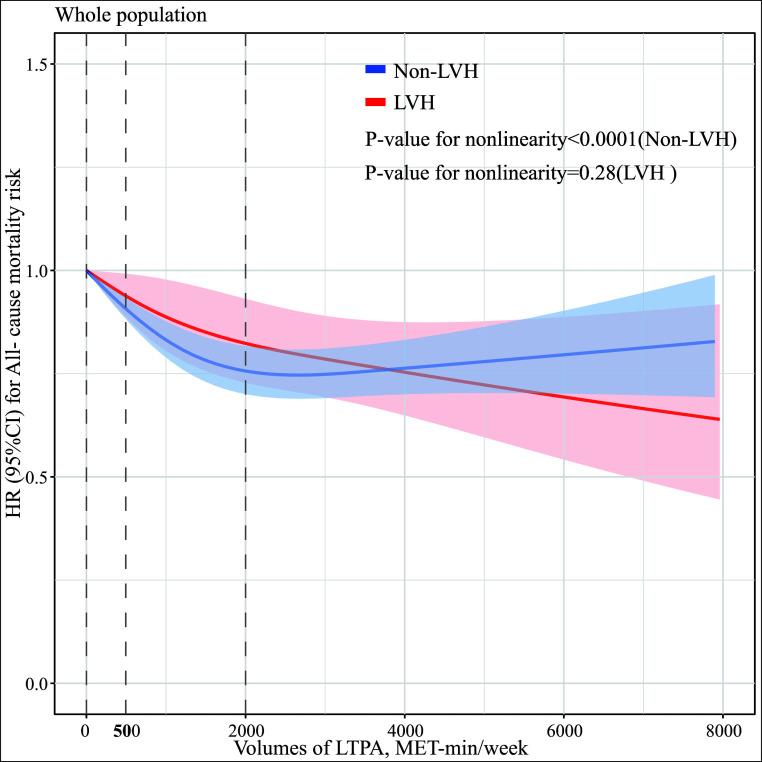
**Dose-response relationship between LTPA (MET-min/week) and mortality with and without LVH.** Restricted cubic spline curves were performed to depict the dose-association between volumes of LTPA and all-cause mortality risk with and without LVH. Models were adjusted for age, sex, BMI, hypertension, dyslipidemia, diabetes, smoking status, drinking, education levels, income levels, sites, and medication use including anti-diabetic, anti-hypertensive, and statins. MET, metabolic equivalent of task; LTPA, leisure-time physical activity; LVH, left ventricular hypertrophy; HR, hazard ratio; CI, confidence interval; BMI, body mass index.

Fig. 3 depicts the dose-response relationship between volumes of LTPA and mortality risk with and without LVH, adjusting for variables as listed previously. The risk of death exhibited an inverse nonlinear association with LTPA in the absence of LVH (*p*-value for non-linearity <0.001), while a linear association in the presence of LVH (*p*-value for non-linearity = 0.28) (Fig. 3).

### 3.4 Stratified and Sensitivity Analyses

In the non-LVH group, moderate and high LTPA were linked with a decreased risk of deaths from all-causes in both males (moderate: HR 0.85; 95% CI: 0.77–0.94, high: HR 0.78; 95% CI: 0.69–0.88) and females (moderate: HR 0.71; 95% CI: 0.61–0.81, high: HR 0.77; 95% CI: 0.64–0.91) (*p*-value for interaction = 0.187). Similar results were also observed across the subgroups by age (<60 and ≥60 years), history of hypertension, or diabetes (**Supplementary Tables 1A–E**).

However, for the individuals with LVH, significant benefits were only observed in high LTPA for females (moderate: HR 1.00; 95% CI: 0.86, 1.17; high: HR 0.67; 95% CI: 0.54, 0.84), rather than males (moderate: HR 0.90; 95% CI: 0.73, 1.12, high: HR 0.86; 95% CI: 0.66, 1.12). Furthermore, in females, the presence of LVH altered the relationships between LTPA and all-cause mortality risk (*p*-value for interaction = 0.006) (Fig. 2). Regarding cardiovascular mortality and other outcomes, the health benefits of LTPA across subgroups were similar (although not all reached the statistical threshold for significance) (**Supplementary Tables 1A–E**).

In sensitivity analyses (**Supplementary Tables 2,3**), the results for all risk estimates were similar when deaths in the first six months of follow-up were excluded, indicating that the reverse causality between LTPA volumes and mortality risk was minor.

## 4. Discussion

In this study involving a large cohort of high CVD risk individuals with or without LVH, we identified significant associations between moderate and high volumes of LTPA and the risk of deaths from all causes or CVD. The correlation patterns appeared distinct between those with LVH and those without. Overall, the associations were not modified by the presence of LVH, despite high LTPA volumes being linked with increased LVMI; while interestingly, the modification of LVH was significant in females regarding all-cause mortality risk.

Both the LVH and LVH-free populations may derive some benefit from moderate and high LTPA, although the dose-relationship of LTPA and mortality risk in the LVH group differs from that of the general population. Our finding was consistent with the results from a prior study including 3078 of the general population, which showed that moderate and high LTPA were linked with a decreased risk of death both in individuals with normal blood pressure and hypertensive patients with a high prevalence of LVH [23]. Moreover, in our study, we extended their result by illustrating the dose-relationship between LTPA and mortality risk in the LVH and non-LVH populations, providing additional evidence that patients with LVH should engage in sufficient volumes of regular physical activity. Notably, in our large-scale study, information on medication and intensity and duration of LTPA was collected, and the latter was quantified as MET-min/week according to the intensity and duration of certain activities, making the results more robust [24]. And this could address the shortcomings of a previous study regarding its small sample size and lack of information on antihypertensive medication information. Additionally, our study focuses on populations at high cardiovascular risk without established CVD and provides fresh insights into the influence of physical exercise on outcomes for this specific group, which had not been accurately characterized in earlier research [23].

High LTPA was more effective than moderate and low LTPA in lowering all-cause mortality risk in the population with LVH. A possible explanation is that high volume LTPA may involve cardiac adaptations which could improve prognosis. Previous studies have demonstrated that long-term strenuous exercise could result in detrimental heart remodeling, including cardiac enlargement, inappropriate myocardial thickening and other abnormalities of diastolic dysfunction [9, 10, 11]. However, these studies focused mostly on athletics and certain specific trainings. Our study indicates that even high volumes of daily physical activity would not increase the risk of mortality in non-athletes, and the increase in volume may be associated with cardiac fitness. Similarly, it is plausible to hypothesize that inappropriate remodeling of the heart may be associated more with the intensity than with the volumes of LTPA. Previous studies have observed that LVH regression could be achieved by pharmacological treatment [25, 26] and modest physical activity (PA) [27, 28], which is consistent with our findings. Additionally, we observed a significantly lower LVMI at about 2000 MET-min/week of LTPA volumes across the subgroups of a high cardiovascular risk population, despite the fact that increased levels of physical activity or exercises were related to a rise in LV mass in patients without LVH at baseline and Asian athletes [11, 23, 29]. The different designs of the study populations might contribute to this contrast. Individuals with high CVD risk factors may be in a different risk category. Therefore, LTPA may have a different effect on their left ventricular mass.

Interestingly, the modification of LVH between LTPA and mortality risk was pronounced in the female population. The PA levels in females were found to be different from those in males both in the healthy and CVD population [30, 31]. Although the precise underlying processes have not been identified, one possible explanation could be females’ particular LV anatomy and high sensitivity to the advantages of LTPA. Females have smaller hearts, which makes the LV more sensitive to myocardial fibrosis [32, 33]. In contrast to males, females tended to have poorer glucose absorption and utilization, higher fatty acid intake and metabolic inefficiency [34], and increased vagal tone due to sex hormones [35]. Additionally, myocardial fibrosis [36], myocardial substrate [37, 38], and autonomic nervous system [39] were the targets of PA on prognosis, which may strengthen the modification of LVH regarding the benefits of LTPA on mortality risk. Overall, the modification of LVH was not significant in the overall population regarding the effects of LTPA on risk of deaths, which could be partly explained by the increased LTPA influence on deaths through its beneficial effect on improving vascular endothelial function [40], lowering blood pressure levels [41], increasing insulin sensitivity, and decreasing CVD risk profiles [42]. These positive effects of LTPA on other established risk factors might affect their interaction, rather than LTPA itself. Nonetheless, the reason for the observed modification is likely to be multi-factorial, which merits further evaluation.

Our study has several clinical implications. First, this study provides new insights into the preventive effects of moderate to high LTPA in LVH patients with high cardiovascular risk. Second, it demonstrates that individuals with LVH tend to acquire lower LTPA levels. In addition to being typically older and suffering from various comorbidities, their myocardial dysfunction impairs their physical performance. Nonetheless, clinicians should emphasize to their patients the value of an active lifestyle. The patients should be encouraged to increase their regular LTPA volume; above 2000 MET-min/week could be considered an applicable requirement for LVH individuals for reducing mortality risk, while volumes above 500 MET-min/week could be beneficial for those without LVH. Third, and more importantly, women are more likely to have LVH, and a regular high LTPA is required to attenuate the mortality risk in LVH individuals. It is probable that women will experience notable advantages, potentially resulting in a decrease in the utilisation of alternative treatments such as pharmaceutical interventions.

Limitations of this study should be acknowledged. First, LTPA was self-reported and whether the LTPA levels changed during the follow-up period was unknown. A large-scale study, however, had verified the accuracy of the PA-related questions [17]. Therefore, we made an assumption that the recollection bias could be minimal. Second, despite rigorous statistical adjustments, it is not possible to exclude the presence of unadjusted confounding factors, such as cardiorespiratory fitness, which was linked to a better prognosis [43]. Notwithstanding these constraints, the majority of participant variables deemed pertinent to mortality risk were incorporated into the models. The findings were robust in sensitivity analysis, thereby enhancing the credibility of the conclusion. However, limited statistical power was observed in some subgroups due to an inadequate sample size.

## 5. Conclusions

In conclusion, our findings indicated that LVH does not significantly modify the link between LTPA and mortality risk in the overall high cardiovascular risk population. However, the presence of LVH altered this correlation in females regarding the all-cause mortality risk.

## Data Availability

The China Patient-centered Evaluative Assessment of Cardiac Events Million Persons Project (China PEACE MPP) data that supported this study are restricted and not publically available. Data are available upon reasonable request and with China PEACE MPP authorization. China PEACE MPP grants conditional data access to qualified researchers with valid requests. Please contact http://cvd-project@nccd.org.cn to seek approval for data access.

## Abbreviations

BMI, body mass index; BSA, body surface area; CVD, cardiovascular disease; China PEACE MPP, China Patient-centered Evaluative Assessment of Cardiac Events Million Persons Project; CI, confidence interval; DBP, diastolic blood pressure; FBG, fasting blood glucose; HR, hazard ratio; ICD-10, International Classification of Diseases, 10th edition; LTPA, leisure-time physical activity; LVM, left ventricular mass; LVMI, left ventricular mass index; LVH, left ventricular hypertrophy; MET, metabolic equivalent of task; PA, physical activity; SD, standard deviation; SBP, systolic blood pressure.

## Supplementary Material

Supplementary material associated with this article can be found, in the online version, at https://doi.org/10.31083/j.rcm2410285.
