# Supplementary material for: Association of Leisure-Time Physical Activity and Mortality Risk in High Cardiovascular Risk Population with and without Left Ventricular Hypertrophy
Source: Rev Cardiovasc Med. 2023 Oct 8;24(10):285. doi: 10.31083/j.rcm2410285 (PMC11273135; doi:10.31083/j.rcm2410285)
Supplement: Supplementary file 1 [file 2153-8174-24-10-285-s1.zip › RCM17883-Supplementary Material-proof.docx]

## Supplementary Materials

**Supplementary Files**

**[Supplementary Methods 3](#_Toc31153)**

[Section 1. Study design and follow-up information of China Patient-centered Evaluative Assessment of Cardiac Events (PEACE) Million Persons Project (MPP) 3](#_Toc25143)

[Section 2. The 2019 CVD risk charts for East Asia by WHO 4](#_Toc8148)

**[Supplementary Results 6](#_Toc22109)**

[Supplementary Fig.1. Flowchart of study participant enrollment 6](#_Toc9685)

[Supplementary Fig.2. Kaplan-Meier curves for mortality risk during follow up according to Leisure-time physical activity and LVH. 7](#_Toc23201)

[Supplementary Table 1. Subgroup analyses of leisure-time physical activity and the risk of all- cause and cardiovascular mortality stratified by the presence of LVH 8](#_Toc7507)

[Supplementary Table 2. Sensitivity analyses of leisure-time physical activity and the risk of mortality stratified by the presence of LVH 18](#_Toc4584)

[Supplementary Table 3. Sensitivity analyses of leisure-time physical activity and the risk of mortality stratified by the presence of LVH in females 19](#_Toc7770)

**Supplementary Methods**

**Section 1. Study** **design** **and** **follow-up** **information** **of** **China** **Patient-centered** **Evaluative** **Assessment** **of** **Cardiac** **Events** **(PEACE)** **Million** **Persons** **Project** **(MPP)**

The China Patient-centered Evaluative Assessment of Cardiac Events (PEACE) Million People Project (MPP) identified permanent inhabitants aged 35 to 75 from 349 rural counties or urban districts (210 rural counties, 139 urban districts) in 31 mainland provinces between August 2014 and December 2021. The project sites-rural counties and urban districts-were chosen based on their geographic positions within each province, the number of inhabitants, the distribution of minority ethnicities, the quality of sickness and death records, and the local capacity to support the research. Provincial coordinating office staff collected basic information (geographic information, economic development, population size, and minority ethnicity distribution) about the selected sites in their province, reported it to the national coordinating office, and discussed it to determine the study sites. Each site picked eight or nine towns or subdistricts based on population size, stability (no substantial population change), local staff commitment, and screening capability. Each town or subdistrict health institution has first screening stations.

China-PEACE MPP links cohort data to the National Mortality Surveillance System and Vital Registration of China Center for Disease Control and Prevention (CDC), which covers urban and rural areas in all 31 mainland Chinese provinces. This allows passive mortality follow-up for each cohort participant. Health care institutions report death data in near real-time, which is checked annually with local residential records and health insurance data. The ninth International Statistical Classification of Diseases codes death causes (ICD-10).

Active follow-up visits, which include questionnaires, physical exams, lab tests, and imaging scans, are recommended for high-risk CVD patients. Participants with extremely high risk (i.e., a history of major cardiovascular events or a predicted risk of CVD in 10 years 20%) have annual face-to-face follow-ups, while those with moderately high risk (severely elevated blood pressure or LDL) have biannual follow-ups with telephone follow-up in between.

**Section 2. The 2019 CVD risk charts for East Asia by WHO**


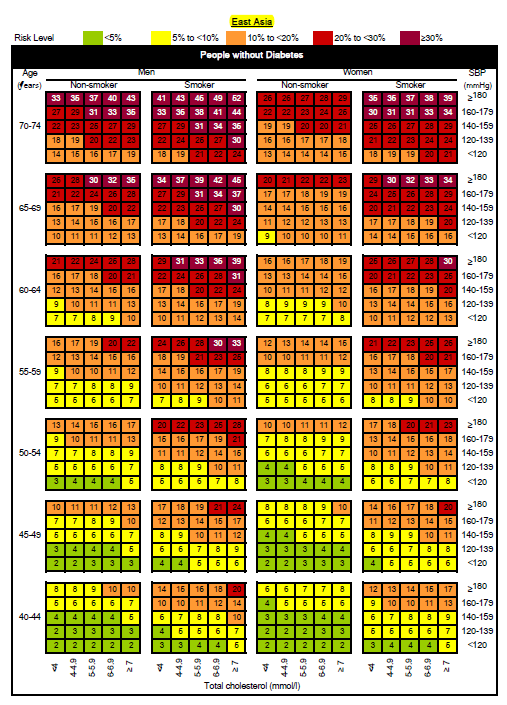


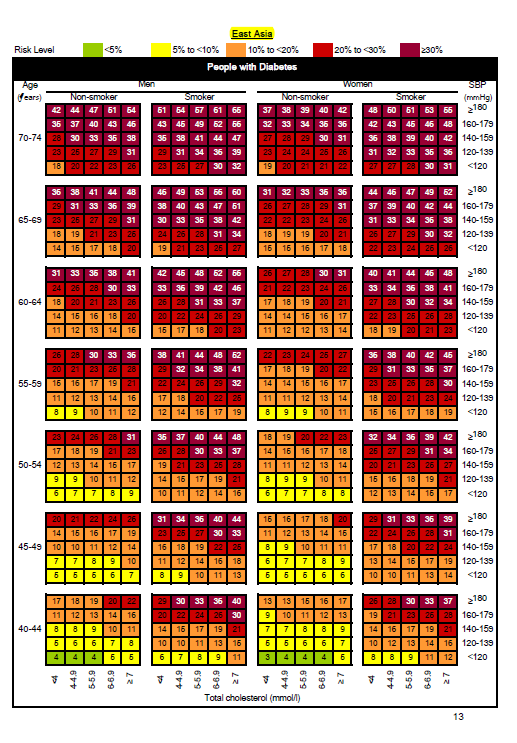


**Supplementary Results**

**Supplementary Fig.1. Flowchart of study participant enrollment**


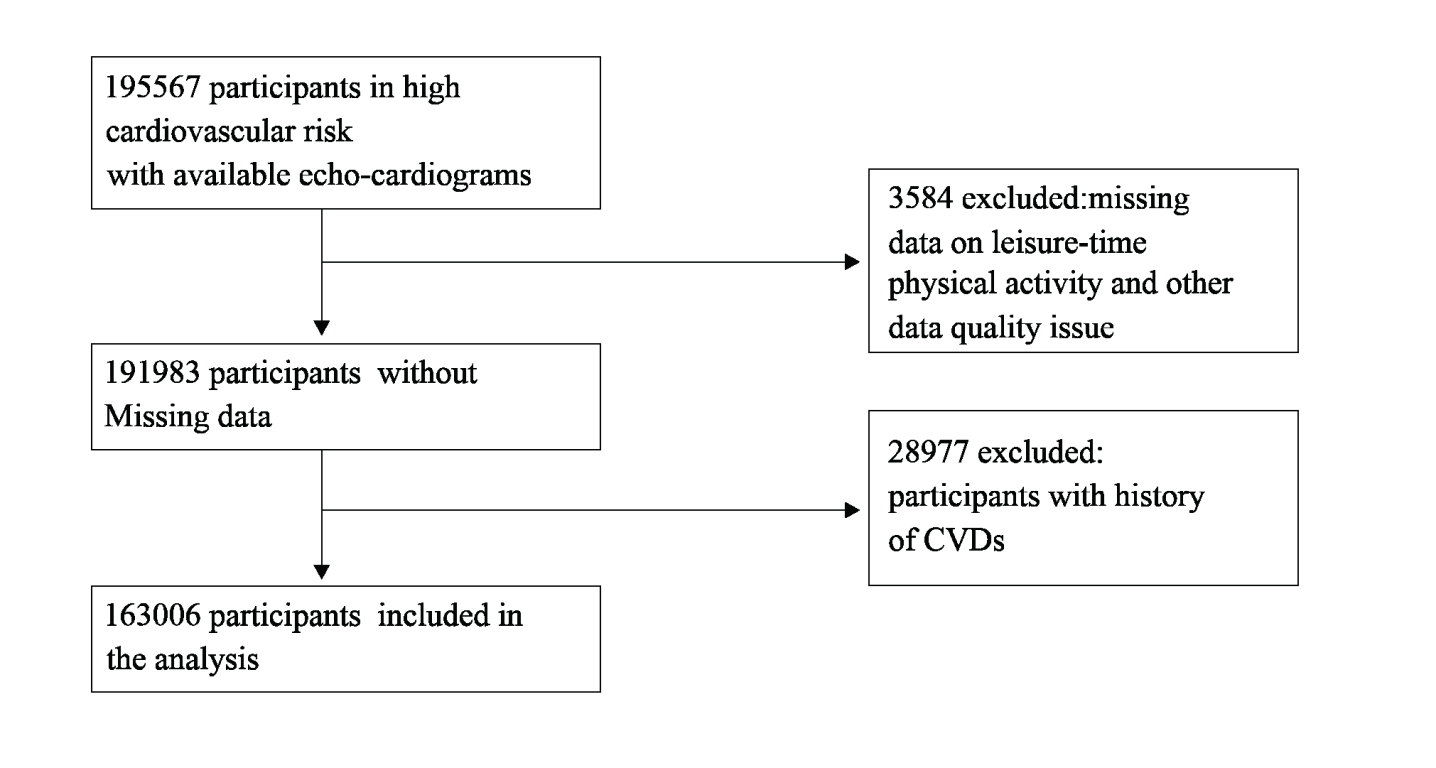


**Supplementary** **Fig.2.** **Kaplan-Meier** **curves** **for** **mortality** **risk** **during** **follow** **up** **according** **to** **Leisure-time** **physical** **activity** **and** **LVH.**


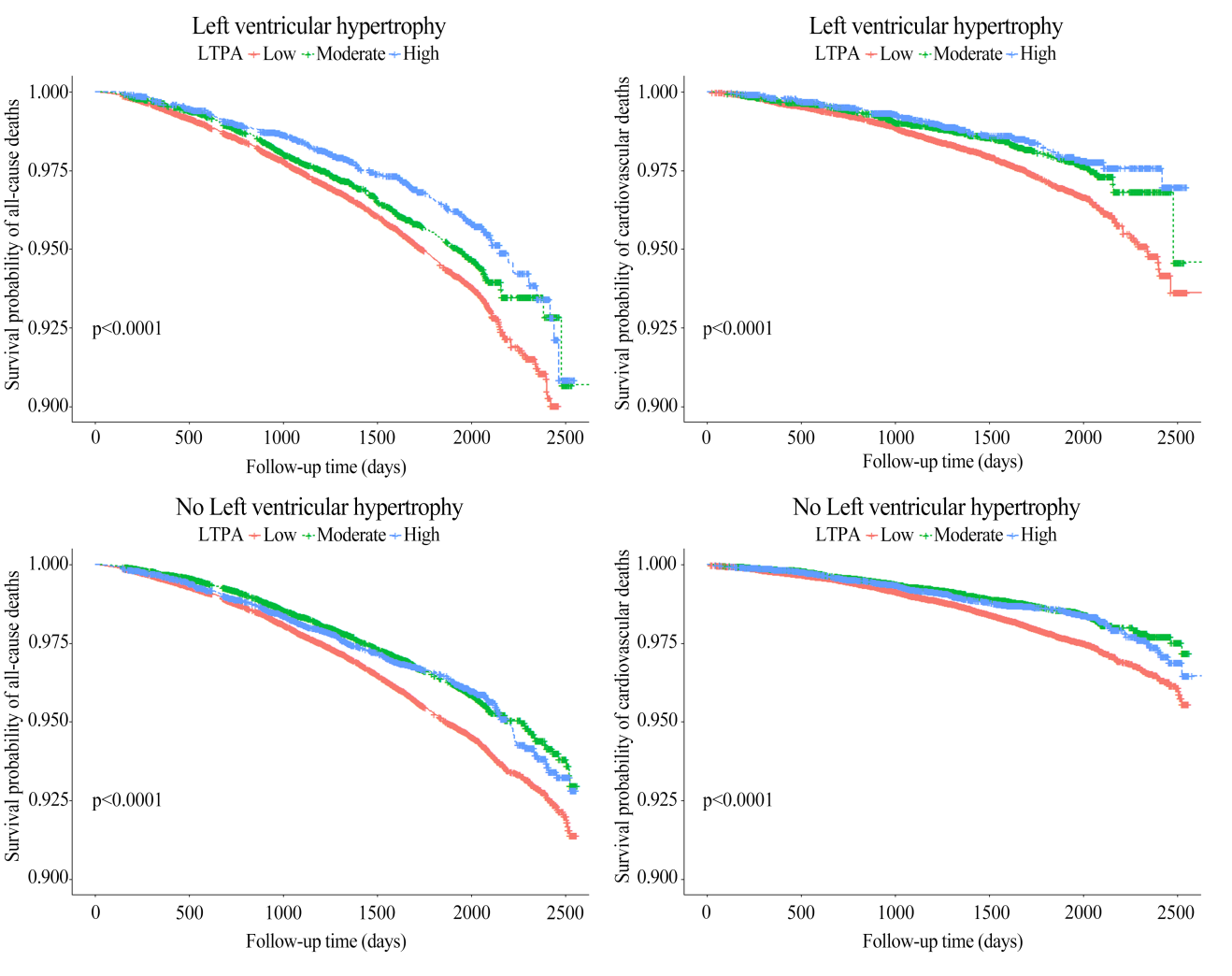


**Fig.** **S2.** **Kaplan-Meier** **curves** **for** **mortality** **risk** **during** **follow** **up** **according** **to** **Leisure-time** **physical** **activity** **and** **LVH**. a, LTPA and all-cause deaths in LVH; b, LTPA and cardiovascular deaths in LVH; c, LTPA and all-cause deaths in Non-LVH; d, LTPA and cardiovascular deaths in Non-LVH. Abbreviations: LTPA, Leisure-time physical activity; LVH, Left ventricular hypertrophy.

**Supplementary** **Table** **1.** **Subgroup** **analyses** **of** **leisure-time** **physical** **activity** **and** **the** **risk** **of** **all-** **cause** **and** **cardiovascular** **mortality** **stratified** **by the** **presence** **of** **LVH**

**A1.** **Leisure-time** **physical** **activity** **and** **the** **risk** **of** **all-** **cause** **mortality** **stratified** **by** **the** **presence** **of** **LVH** **in** **male** **and** **female**

| Volumes of  leisure-time physical activity (MET-min/week) | Male | | Female | |
| --- | --- | --- | --- | --- |
|  | Rates, % (N) | Adjusted  HR (95% CI) * | Rates, % (N) | Adjusted  HR (95% CI) * |
| **Non-** **LVH** | 4.92%(3128) |  | 2.60% (1473) |  |
| Low(<500) | 5.37% (2222) | Reference | 2.93% (1064) | Reference |
| Moderate(500- 1999) | 4.05% (580) | 0.85 (0.77-0.94) | 1.91% (257) | 0.71 (0.61-0.81) |
| High(≥2000) | 4. 15% (326) | 0.78 (0.69-0.88) | 2. 19% (152) | 0.77 (0.62-0.85) |
| **LVH** | 7.59% (710) |  | 3.81% (1275) |  |
| Low(<500) | 8.06% (529) | Reference | 4. 13% (958) | Reference |
| Moderate(500- 1999) | 6.46% (112) | 0.90 (0.73- 1. 12) | 3.42% (224) | 1.00 (0.86- 1. 17) |
| High(≥2000) | 6.58% (69) | 0.86 (0.66- 1. 12) | 2.52% (93) | 0.67 (0.54-0.84) |

The interaction effect of LTPA and LVH was *P* for interaction=0.765 in males; *P* for interaction=0.006 in females

* Models were adjusted for age, BMI, hypertension, dyslipidemia, diabetes, smoking status, drinking, education levels, income levels, sites, and medication use including anti-diabetic, anti-hypertensive, and statins.

**A2.** **Leisure-time** **physical** **activity** **and** **the** **risk** **of** **cardiovascular** **mortality** **stratified** **by** **the** **presence** **of** **LVH** **in** **male** **and** **female** **in** **male** **and** **female**

| Volumes of  leisure-time physical activity (MET-min/week) | Male | | Female | |
| --- | --- | --- | --- | --- |
|  | Rates, % (N) | Adjusted  HR (95% CI) * | Rates, % (N) | Adjusted  HR (95% CI) * |
| **Non-** **LVH** | 2. 12%(1349) |  | 1. 17% (662) |  |
| Low(<500) | 2.39% (989) | Reference | 1.37% (496) | Reference |
| Moderate(500- 1999) | 1.63% (234) | 0.79 (0.68-0.91) | 0.72% (97) | 0.58 (0.46-0.72) |
| High(≥2000) | 1.60% (126) | 0.68 (0.56-0.82) | 0.99% (69) | 0.76 (0.58-0.99) |
| **LVH** | 3.86% (361) |  | 1.95% (652) |  |
| Low(<500) | 4.20% (276) | Reference | 2.20% (509) | Reference |
| Moderate(500- 1999) | 3.23% (56) | 0.89 (0.65- 1.21) | 1.42% (93) | 0.81 (0.65- 1.02) |
| High(≥2000) | 2.76% (29) | 0.66 (0.44- 1.00) | 1.35% (50) | 0.69 (0.51-0.94) |

The interaction effect of LTPA and LVH was *P* for interaction=0.716 in males; *P* for interaction=0.531 in females

* Models were adjusted for age, BMI, hypertension, dyslipidemia, diabetes, smoking status, drinking, education levels, income levels, sites, and medication use including anti-diabetic, anti-hypertensive, and statins.

**B1.** **Leisure-time** **physical** **activity** **and** **the** **risk** **of** **all-** **cause** **mortality** **stratified** **by** **the** **presence** **of** **LVH** **in** **age≥60** **years** **and** **age<60** **years**

| Volumes of  leisure-time physical activity (MET-min/week) | Age ≥60 years | | Age <60 years | |
| --- | --- | --- | --- | --- |
|  | Rates, % (N) | Adjusted  HR (95% CI) * | Rates, % (N) | Adjusted  HR (95% CI) * |
| **Non-** **LVH** | 4.99%(3700) |  | 1.96% (901) |  |
| Low(<500) | 5.67% (2617) | Reference | 2. 12% (669) | Reference |
| Moderate(500- 1999) | 3.75% (674) | 0.80 (0.73-0.88) | 1.66% (163) | 0.98 (0.82- 1. 17) |
| High(≥2000) | 4.05% (409) | 0.80 (0.72-0.89) | 1.47% (69) | 0.85 (0.66- 1. 10) |
| **LVH** | 5.41% (1661) |  | 2.68% (324) |  |
| Low(<500) | 5.94% (1242) | Reference | 2.77% (245) | Reference |
| Moderate(500- 1999) | 4.58% (283) | 0.97 (0.84- 1. 11) | 2.52% (53) | 1.00 (0.84- 1.55) |
| High(≥2000) | 3.76% (136) | 0.71 (0.59-0.85) | 2.31% (26) | 1. 10 (0.67- 1.54) |

The interaction effect of LTPA and LVH was *P* for interaction=0.055 in age≥60 years; *P* for interaction=0.613 in age≥60 years

* Models were adjusted for sex, BMI, hypertension, dyslipidemia, diabetes, smoking status, drinking, education levels, income levels, sites, and medication use including anti-diabetic, anti-hypertensive, and statins.

**B2.** **Leisure-time** **physical** **activity** **and** **the** **risk** **of** **cardiovascular** **mortality** **stratified** **by** **the** **presence** **of** **LVH** **in** **age≥60** **years** **and** **age<60** **years** **in** **age≥60** **years** **and** **age<60** **years**

| Volumes of  leisure-time physical activity (MET-min/week) | Age ≥60 years | | Age <60 years | |
| --- | --- | --- | --- | --- |
|  | Rates, % (N) | Adjusted  HR (95% CI) * | Rates, % (N) | Adjusted  HR (95% CI) * |
| **Non-** **LVH** | 2. 16% (1600) |  | 0.89% (411) |  |
| Low(<500) | 2.55% (1177) | Reference | 0.98% (308) | Reference |
| Moderate(500- 1999) | 1.46% (262) | 0.58 (0.46-0.72) | 0.70% (69) | 0.90 (0.69- 1. 18) |
| High(≥2000) | 1.59% (161) | 0.76 (0.58-0.99) | 0.72% (34) | 0.90 (0.62- 1.29) |
| **LVH** | 2.78% (853) |  | 1.32% (160) |  |
| Low(<500) | 3. 17% (663) | Reference | 1.38% (122) | Reference |
| Moderate(500- 1999) | 1.99% (123) | 0.81 (0.65- 1.02) | 1.24% (26) | 1.20 (0.77- 1.88) |
| High(≥2000) | 1.85% (67) | 0.69 (0.51-0.94) | 1.07% (12) | 0.94 (0.50- 1.75) |

The interaction effect of LTPA and LVH was *P* for interaction=0.639 in age≥60 years; *P* for interaction=0.665 in age≥60 years

* Models were adjusted for sex, BMI, hypertension, dyslipidemia, diabetes, smoking status, drinking, education levels, income levels, sites, and medication use including anti-diabetic, anti-hypertensive, and statins.

**C1.** **Leisure-time** **physical** **activity** **and** **the** **risk** **of** **all-** **cause** **mortality** **stratified** **by** **the** **presence** **of** **LVH** **in** **hypertension** **and** **non-hypertension**

| Volumes of  leisure-time physical activity (MET-min/week) | Hypertension | | Non-hypertension | |
| --- | --- | --- | --- | --- |
|  | Rates, % (N) | Adjusted  HR (95% CI) * | Rates, % (N) | Adjusted  HR (95% CI) * |
| **Non-** **LVH** | 3.87% (4096) |  | 3.49% (505) |  |
| Low (<500) | 4.27% (2944) | Reference | 3.96% (342) | Reference |
| Moderate (500- 1999) | 3. 10% (743) | 0.81 (0.74-0.88) | 2.49% (94) | 0.72 (0.57-0.92) |
| High (≥2000) | 3.21% (409) | 0.75 (0.68-0.84) | 3.34% (69) | 0.91 (0.69- 1.20) |
| **LVH** | 4.72% (1885) |  | 3.50% (100) |  |
| Low (<500) | 5.07% (1414) | Reference | 3.98% (73) | Reference |
| Moderate (500- 1999) | 4. 13% (316) | 0.95 (0.83- 1.08) | 3. 19% (20) | 1. 17 (0.69- 1.99) |
| High (≥2000) | 3.56% (155) | 0.74 (0.62-0.88) | 1.77% (7) | 0.49 (0.22- 1. 10) |

The interaction effect of LTPA and LVH was *P* for interaction=0.231 in hypertension; *P* for interaction=0.083 in non- hypertension

* Models were adjusted for age, sex, BMI, dyslipidemia, diabetes, smoking status, drinking, education levels, income levels, sites, and medication use including anti-diabetic, anti-hypertensive, and statins.

**C2.** **Leisure-time** **physical** **activity** **and** **the** **risk** **of** **cardiovascular** **mortality** **stratified** **by** **the** **presence** **of** **LVH** **in** **hypertension** **and** **non-** **hypertension**

| Volumes of  leisure-time physical activity (MET-min/week) | Hypertension | | Non- hypertension | |
| --- | --- | --- | --- | --- |
|  | Rates, % (N) | Adjusted  HR (95% CI) * | Rates, % (N) | Adjusted  HR (95% CI) * |
| **Non-** **LVH** | 1.75%(1853) |  | 1.09% (158) |  |
| Low(<500) | 1.99%(1372) | Reference | 1.31% (113) | Reference |
| Moderate(500- 1999) | 1.27% (305) | 0.71 (0.62-0.81) | 0.69% (26) | 0.66 (0.42- 1.04) |
| High(≥2000) | 1.38% (176) | 0.68 (0.58-0.80) | 0.92% (19) | 0.84 (0.49- 1.43) |
| **LVH** | 3.50% (973) |  | 1.40% (40) |  |
| Low(<500) | 2.71% (755) | Reference | 1.64% (30) | Reference |
| Moderate(500- 1999) | 1.85% (142) | 0.84 (0.69- 1.00) | 1. 12% (7) | 1. 11 (0.49-2.51) |
| High(≥2000) | 1.75% (76) | 0.69 (0.54-0.88) | 0.76% (3) | 0.47 (0. 13- 1.78) |

The interaction effect of LTPA and LVH was *P* for interaction=0.670 in hypertension; *P* for interaction=0.433 in non- hypertension

* Models were adjusted for age, sex, BMI, dyslipidemia, diabetes, smoking status, drinking, education levels, income levels, sites, and medication use including anti-diabetic, anti-hypertensive, and statins.

**D1.** **Leisure-time** **physical** **activity** **and** **the** **risk** **of** **all-** **cause** **mortality** **stratified** **by** **the** **presence** **of** **LVH** **in** **Diabetes** **and** **non-** **Diabetes**

| Volumes of  leisure-time physical activity (MET-min/week) | Diabetes | | Non- diabetes | |
| --- | --- | --- | --- | --- |
|  | Rates, % (N) | Adjusted  HR (95% CI) * | Rates, % (N) | Adjusted  HR (95% CI) * |
| **Non-** **LVH** | 3.99%(1695) |  | 3.74% (2906) |  |
| Low(<500) | 4.27% (1140) | Reference | 4. 17% (2146) | Reference |
| Moderate(500- 1999) | 3.43% (371) | 0.86 (0.76-0.97) | 2.75% (466) | 0.75 (0.68-0.84) |
| High(≥2000) | 3.32% (184) | 0.77 (0.66-0.91) | 3. 17% (294) | 0.77 (0.68-0.88) |
| **LVH** | 5.26% (767) |  | 4.32% (1218) |  |
| Low(<500) | 5.65% (556) | Reference | 4.68% (931) | Reference |
| Moderate(500- 1999) | 4.65% (142) | 0.91 (0.75- 1. 10) | 3.71% (194) | 1.00 (0.85- 1. 18) |
| High(≥2000) | 4.08% (69) | 0.72 (0.56-0.94) | 3.05% (93) | 0.73 (0.59-0.92) |

The interaction effect of LTPA and LVH was *P* for interaction=0.779 in Diabetes; *P* for interaction=0.057 in non- Diabetes

* Models were adjusted for age, sex, BMI, hypertension, dyslipidemia,smoking status, drinking, education levels, income levels, sites, and medication use including anti-diabetic, anti-hypertensive, and statins.

**D2.** **Leisure-time** **physical** **activity** **and** **the** **risk** **of** **cardiovascular** **mortality** **stratified** **by** **the** **presence** **of** **LVH** **in** **Diabetes** **and** **non-** **Diabetes**

| Volumes of  leisure-time physical activity (MET-min/week) | Diabetes | | Non- diabetes | |
| --- | --- | --- | --- | --- |
|  | Rates, % (N) | Adjusted  HR (95% CI) * | Rates, % (N) | Adjusted  HR (95% CI) * |
| **Non-** **LVH** | 1.77%(753) |  | 1.62%(1258) |  |
| Low(<500) | 2.04%(534) | Reference | 1.85%(951) | Reference |
| Moderate(500- 1999) | 1.32% (143) | 0.71 (0.59-0.86) | 1. 11% (188) | 0.69 (0.59-0.82) |
| High(≥2000) | 1.37% (76) | 0.69 (0.54-0.88) | 1.28% (119) | 0.69 (0.57-0.85) |
| **LVH** | 3.50% (393) |  | 2.20% (620) |  |
| Low(<500) | 2.99% (294) | Reference | 2.47% (491) | Reference |
| Moderate(500- 1999) | 2. 13% (65) | 0.85 (0.65- 1. 11) | 1.61% (84) | 0.84 (0.66- 1.08) |
| High(≥2000) | 2.01% (34) | 0.69 (0.47- 1.00) | 1.47% (45) | 0.67 (0.48-0.92) |

The interaction effect of LTPA and LVH was *P* for interaction=0.651 in Diabetes; *P* for interaction=0.619 in non- Diabetes

* Models were adjusted for age, sex, BMI, hypertension, dyslipidemia, smoking status, drinking, education levels, income levels, sites, and medication use including anti-diabetic, anti-hypertensive, and statins.

**E1.** **Leisure-time** **physical** **activity** **and** **the** **risk** **of** **all-** **cause** **mortality** **stratified** **by** **the** **presence** **of** **LVH** **in** **Obesity** **and** **non-** **Obesity**

| Volumes of  leisure-time physical activity (MET-min/week) | Obesity | | Non- Obesity | |
| --- | --- | --- | --- | --- |
|  | Rates, % (N) | Adjusted  HR (95% CI) * | Rates, % (N) | Adjusted  HR (95% CI) * |
| **Non- LVH** | 3.17%(1727) |  | 4.37%(2874) |  |
| Low(<500) | 3.40% (1149) | Reference | 4.88%(2137) | Reference |
| Moderate(500- 1999) | 2.77% (380) | 0.82 (0.73-0.93) | 3.26%(457) | 0.78 (0.70-0.87) |
| High(≥2000) | 2.86% (198) | 0.77 (0.66-0.90) | 3.55%(280) | 0.78 (0.69-0.89) |
| **LVH** | 4.08% (522) |  | 4.88%( 1463) |  |
| Low(<500) | 4. 17% (356) | Reference | 5.33%( 1131) | Reference |
| Moderate(500- 1999) | 4.07% (114) | 1.05 (0.84- 1.31) | 4.05%(222) | 0.92 (0.79- 1.07) |
| High(≥2000) | 3.51% (52) | 0.85 (0.62- 1. 15) | 3.37%( 110) | 0.69 (0.56-0.84) |

The interaction effect of LTPA and LVH was *P* for interaction=0.314 in Obesity; *P* for interaction=0. 124 in non- Obesity

* Models were adjusted for age, sex, hypertension, dyslipidemia, diabetes, smoking status, drinking, education levels, income levels, sites, and medication use including anti-diabetic, anti-hypertensive, and statins.

**E2.** **Leisure-time** **physical** **activity** **and** **the** **risk** **of** **cardiovascular** **mortality** **stratified** **by** **the** **presence** **of** **LVH** **in** **Obesity** **and** **non-** **Obesity**

| Volumes of  leisure-time physical activity (MET-min/week) | Obesity | | Non-Obesity | |
| --- | --- | --- | --- | --- |
|  | Rates, % (N) | Adjusted  HR (95% CI) * | Rates, % (N) | Adjusted  HR (95% CI) * |
| **Non-** **LVH** | 1.38%(753) |  | 1.91%(1258) |  |
| Low (<500) | 1.53%(518) | Reference | 2.21%(967) | Reference |
| Moderate (500- 1999) | 1. 11% (152) | 0.76. (0.63-0.91) | 1.28% (179) | 0.67 (0.57-0.80) |
| High (≥2000) | 1.20% (83) | 0.72 (0.57-0.92) | 1.42% (112) | 0.68 (0.55-0.84) |
| **LVH** | 2.00% (256) |  | 2.53% (757) |  |
| Low (<500) | 2.02% (172) | Reference | 2.89% (613) | Reference |
| Moderate (500- 1999) | 2.07% (58) | 1.22 (0.88- 1.70) | 1.66% (91) | 0.72 (0.58-0.91) |
| High (≥2000) | 1.76% (26) | 0.90 (0.58- 1.41) | 1.62% (53) | 0.62 (0.46-0.83) |

The interaction effect of LTPA and LVH was *P* for interaction=0.096 in Obesity; *P* for interaction=0.687 in non- Obesity

* Models were adjusted for age, sex, hypertension, dyslipidemia, diabetes, smoking status, drinking, education levels, income levels, sites, and medication use including anti-diabetic, anti-hypertensive, and statins.

**Supplementary** **Table** **2.** **Sensitivity** **analyses** **of** **leisure-time** **physical** **activity** **and** **the** **risk** **of** **mortality** **stratified** **by** **the** **presence** **of** **LVH**

| Volumes of  leisure-time physical activity (MET-min/week) | All-cause mortality | | | Cardiovascular mortality | | |
| --- | --- | --- | --- | --- | --- | --- |
|  | Rates, % (N) | Unadjusted  HR (95% CI) | Adjusted  HR (95% CI) * | Rates, % (N) | Unadjusted  HR (95% CI) | Adjusted  HR (95% CI)* |
| **Non-** **LVH** | 3.74% (4492) |  |  | 1.62% (1951) |  |  |
| Low(<500) | 4. 13% (3206) | Reference | Reference | 1.86% (1444) | Reference | Reference |
| Moderate(500- 1999) | 2.96% (820) | 0.77 (0.58-0.74) | 0.72 (0.72-0.84) | 1. 15% (318) | 0.65 (0.58-0.74) | 0.71 (0.63-0.81) |
| High(≥2000) | 3. 15% (466) | 0.76 (0.59-0.81) | 0.76 (0.69-0.84) | 1.28% (189) | 0.69 (0.59–0.81) | 0.73 (0.62-0.85) |
| **LVH** | 4.56% (1948) |  |  | 2.33% (995) |  |  |
| Low(<500) | 4.90% (1456) | Reference | Reference | 2.59% (771) | Reference | Reference |
| Moderate(500- 1999) | 4.00% (331) | 0.88 (0.78-0.99) | 0.82 (0.68-0.99) | 1.75% (145) | 0.71 (0.59-0.85) | 0.83 (0.69-0.99) |
| High(≥2000) | 3.39% (161) | 0.67 (0.57-0.79) | 0.73 (0.57-0.93) | 1.67% (79) | 0.63 (0.50-0.79) | 0.72 (0.56-0.91) |

The interaction effect of LTPA and LVH was *P* for interaction=0.074 for all-cause mortality; *P* for interaction=0.563 for cardiovascular mortality mortality.

* Models were adjusted for age, sex, BMI, hypertension, dyslipidemia, diabetes, smoking status, drinking, education levels, income levels, sites, and medication use including anti-diabetic, anti-hypertensive, and statins.

**Supplementary Table 3. Sensitivity analyses of leisure-time physical activity and the risk** **of** **mortality** **stratified** **by** **the** **presence** **of** **LVH in females**

| Volumes of  leisure-time physical activity  (MET-min/week) | All-cause mortality | | CVD mortality | |
| --- | --- | --- | --- | --- |
|  | Rates, % (N) | Adjusted  HR (95% CI) * | Rates, % (N) | Adjusted  HR (95% CI)* |
| **Non- LVH** | 2.55% (1444) |  | 1.14%(648) |  |
| Low (<500) | 2.87% (1040) | Reference | 1.34% (484) | Reference |
| Moderate (500- 1999) | 1.89% (254) | 0.71 (0.62-0.82) | 0.71% (95) | 0.59 (0.47-0.74) |
| High (≥2000) | 2.16% (150) | 0.77 (0.65-0.93) | 0.99% (69) | 0.80 (0.61-1.05) |
| **LVH** | 3.75% (1254) |  | 1.92% (640) |  |
| Low (<500) | 4.06% (941) | Reference | 2.16% (500) | Reference |
| Moderate (500- 1999) | 3.36% (220) | 1.00 (0.86-1.17) | 1.37%(90) | 0.79 (0.63-0.99) |
| High (≥2000) | 2.52% (93) | 0.69 (0.55-0.86) | 1.35% (50) | 0.73 (0.55-1.00) |

The interaction effect of LTPA and LVH was *P* for interaction=0.086 for all-cause mortality; *P* for interaction=0.247 for cardiovascular mortality mortality.

* Models were adjusted for age, BMI, hypertension, dyslipidemia, diabetes, smoking status, drinking, education levels, income levels, sites, and medication use including anti-diabetic, anti-hypertensive, and statins.
