# Supplementary figures and images for: Association of Leisure-Time Physical Activity and Mortality Risk in High Cardiovascular Risk Population with and without Left Ventricular Hypertrophy
Source: Rev Cardiovasc Med. 2023 Oct 8;24(10):285. doi: 10.31083/j.rcm2410285 (PMC11273135; doi:10.31083/j.rcm2410285)

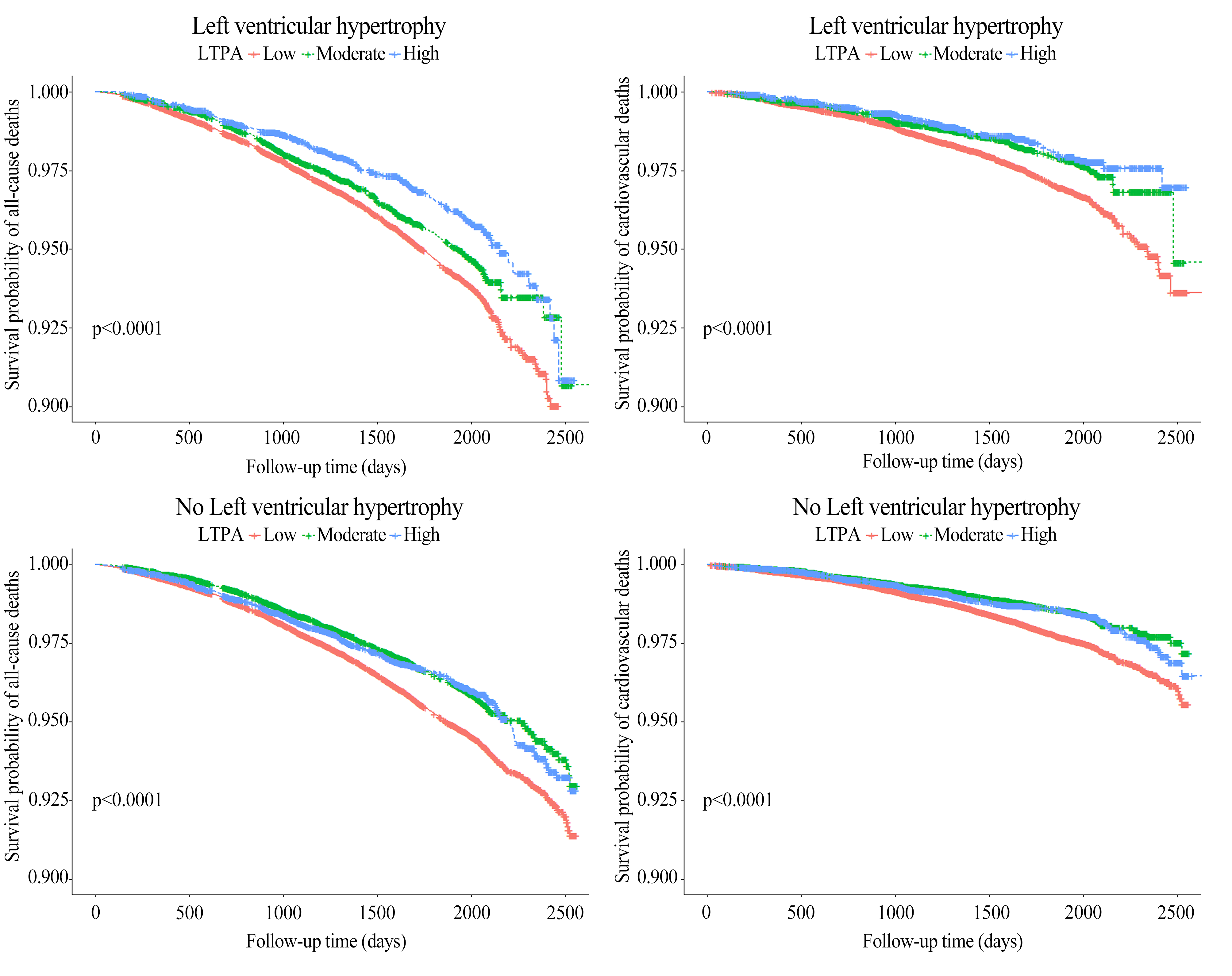

Supplement: Supplementary file 1 [file 2153-8174-24-10-285-s1.zip › Fig.S2.tif]
